# Peak neutralizing and cross-neutralizing antibody levels to human papillomavirus types 6/16/18/31/33/45/52/58 induced by bivalent and quadrivalent HPV vaccines

**DOI:** 10.1038/s41541-020-0165-x

**Published:** 2020-02-14

**Authors:** Filipe Colaço Mariz, Noemi Bender, Devasena Anantharaman, Partha Basu, Neerja Bhatla, Madhavan Radhakrisna Pillai, Priya R. Prabhu, Rengaswamy Sankaranarayanan, Tiina Eriksson, Michael Pawlita, Kristina Prager, Peter Sehr, Tim Waterboer, Martin Müller, Matti Lehtinen

**Affiliations:** 1 Tumorvirus-Specific Vaccination Strategies, Deutsches Krebsforschungszentrum (DKFZ), 69120 Heidelberg, Germany; 2 Infections and Cancer Epidemiology, Deutsches Krebsforschungszentrum (DKFZ), 69120 Heidelberg, Germany; 3 Rajiv Gandhi Centre for Biotechnology, Poojappura, Thiruvananthapuram, 695014 Kerala India; 4 Screening Group, International Agency for Research on Cancer (IARC), 69008 Lyon, France; 5 All India Institute of Medical Sciences, 110029 New Delhi, India; 6 Research Triangle Institute International India, 6th Floor, Pullman Commercial Tower, Aero City, 110037 New Delhi India; 7 Karolinska Institute, 17177 Stockholm, Sweden; 8 EMBL-DKFZ Chemical Biology Core Facility, European Molecular Biology Laboratory, 69117 Heidelberg, Germany

**Keywords:** Cervical cancer, Viral infection

## Abstract

We performed an independent comparison of neutralizing and cross-neutralizing antibody (ab) levels seven months after initiation of three-dose, six-month vaccination schedules with the bivalent and quadrivalent human papillomavirus (HPV) vaccines in adolescent Finnish and Indian females, respectively. We used a semi-automated Pseudovirion-Based Neutralization Assay and observed significantly higher HPV16/18 peak ab-levels in bivalent as compared to quadrivalent vaccine recipients. Bivalent vaccine induced cross-neutralizing HPV31/33/45/52/58 antibodies significantly more frequently and to higher levels than the quadrivalent vaccine. The correlation of bivalent vaccine-induced HPV45 ab-levels with HPV16/18 ab-levels was stronger than that of corresponding quadrivalent vaccine-induced ab-levels, suggesting a qualitatively different cross-reactive response. Our findings on the comparison of the immunogenicity of two HPV vaccine tested in two different populations indicate that further head-to-head studies are warranted.

## Introduction

Differences in the breadth of cross-protective vaccine efficacies (VE) of prophylactic bivalent and quadrivalent human papillomavirus (HPV) virus-like particle (VLP) vaccines against infections with high-risk (hr) HPV types and associated neoplasia are well established. These extend to significantly different vaccine-specific overall efficacies against associated high-grade squamous intraepithelial neoplasia irrespective of HPV type.^1,2^ The considerable overlap, with regard to age, ethnicity and sexual risk-taking behavior of different female target populations in the major clinical phase III trials involving the two vaccines^2,3^, suggests that the differences in efficacy most likely are vaccine-specific.^1–4^

Neutralizing antibodies induced upon VLP vaccination have been suggested to be the primary mechanism in mediating protection from HPV infection.^5^ The impact of HPV cross-neutralizing antibodies in promoting cross-protection against non-vaccine types is, however, uncertain. Independent studies, considering early adolescent girls or HIV-positive individuals, have demonstrated that cross-neutralizing antibodies induced by the bivalent vaccine confer wider cross-neutralizing antibody response and wider protection against cervical hrHPV infections and associated intraepithelial lesions than the quadrivalent vaccine.^6–8^ Also an independent study on vaccine-induced antibody sustainability in adolescent females found that bivalent vaccine-induced total HPV16 and HPV18 L1-VLP binding antibody levels were 5- and 18-fold (respectively) higher than those for the quadrivalent vaccine up to 12 years post vaccination.^9^

The launching of a nonavalent HPV6/11/16/18/31/33/45/52/58 VLP vaccine to the market raises two pivotal questions for public health decision makers in respect to national vaccination programs: (1) how broad is the cross-neutralization ability of the bivalent vaccine-induced antibodies? (2) How sustainable are the quadri/nonavalent vs. bivalent vaccine-induced neutralizing and cross-neutralizing antibodies?

In this report, we particularly address the extent and type-specific pattern of cross-neutralization induced by two different HPV vaccines. Cross-neutralizing antibody titers are always substantially lower than titers against vaccine types. Specifically, we investigated peak (seven months post vaccination) neutralizing antibody titers induced by the bivalent and quadrivalent vaccines to HPV types 6/16/18/31/33/45/52/58 in adolescent Finnish and Indian women, respectively, to reveal the breadth of the cross-neutralizing antibody responses of bivalent versus multivalent vaccines.

## Results

### Neutralizing antibody levels and seroprevalence

**Fig. 1:**
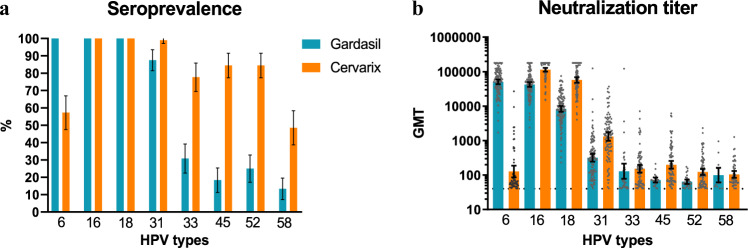
Seropositivity and neutralizing antibody levels induced by the bivalent and quadrivalent vaccines. **a** Percentage of vaccine recipients with neutralizing antibody titers of >40 to vaccine HPV types ((6)/16/18 and non-vaccine HPV types (6)/31/33/45/52/58). Bars indicate boundaries of 95% confidence interval (CI). Sera of quadrivalent (Gardasil, 6/11/16/18) and bivalent (Cervarix, 16/18) vaccine recipients are shown in blue and orange columns, respectively. **b** Neutralizing peak (Month 7) antibody levels (Geometric mean titer, GMT) in neutralization-positive samples. The gray dots represent the EC50 values of one serum. Serum concentrations inhibiting 50% of the PsV infection (EC50 values) were calculated from median of triplicates.

All vaccinated study participants at Month 7 showed neutralizing antibodies to HPV types 16 and 18 (Fig. 1a) shared by both vaccines. Bivalent/Finnish and quadrivalent/Indian vaccine recipients differed in median titers (166,681 (5,373 IU/ml) versus 46,400 (1,495 IU/ml) for HPV16 and 57,369 (1,599 IU/ml) versus 8859 (247 IU/ml) for HPV18) and percentage of sera with titers >180,000 (47% versus 6% for HPV16, and 20% versus 0% for HPV18). Without correction for titers >180,000, geometric mean neutralization titers against HPV16 and HPV18 were, respectively, 2.7- and 6.9-fold higher in the bivalent/Finnish vaccine recipients than in the quadrivalent/Indian vaccine recipients (Fig. 1b). Notably, the vaccine-induced median HPV16 antibody titer of 166,681 in the bivalent/Finnish vaccine recipients was above the upper 95% confidence limit of the HPV16 GMT in the quadrivalent/Indian vaccine recipients.

Antibodies cross-neutralizing HPV45 and HPV52, respectively, were found more frequently (18% vs 84%, and 25% vs 84%) (Fig. 1a) and at 2- to 3-fold higher titers in the bivalent/Finnish vaccine recipients as compared to the quadrivalent/Indian vaccine recipients (Fig. 1b). Although HPV31 seroprevalence in bivalent/Finnish vaccine recipients (99.0%) was not much higher than that observed in quadrivalent/Indian vaccine recipients (87.6%) (Fig. 1a), the cross-neutralizing HPV31 antibody titers induced by the bivalent vaccine were 4.1-fold higher than those induced by the quadrivalent vaccine (Fig. 1b). Contrarily, the seroprevalence observed to HPV33 and HPV58 in bivalent/Finnish vaccine recipients was 2.4- to 3.5-fold higher than in quadrivalent/Indian vaccine recipients, although cross-neutralizing antibody levels were not much different.

HPV6 is part of the quadrivalent vaccine but absent in the bivalent vaccine. All quadrivalent/Indian vaccine recipient sera neutralized HPV6. In the bivalent/Finnish vaccine recipients we observed cross-neutralization of HPV6 (Fig. 1a) but with a significantly lower prevalence (57.2%). The GMT of quadrivalent vaccine-induced neutralizing HPV6 antibodies was 407-fold higher than that of the cross-neutralizing HPV6 antibodies induced by the bivalent vaccine (Fig. 1b).

**Fig. 2:**
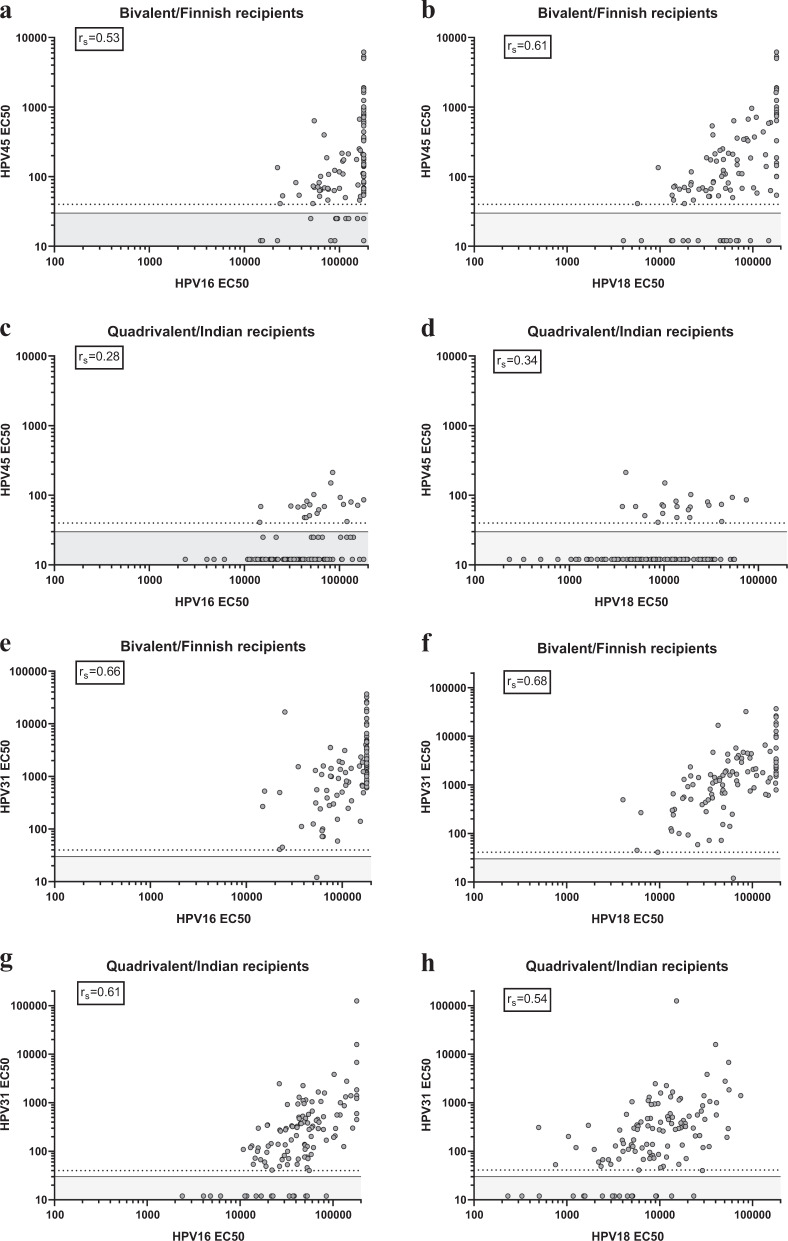
Correlations between neutralizing and cross-neutralizing antibody titers induced by the HPV vaccines. EC50 against HPV16/45 (**a** and **c**), HPV18/45 (**b** and **d**), HPV16/31 (**i** and **g**) and HPV18/31 (**f** and **h**) induced in the bivalent/Finnish and the quadrivalent/Indian vaccine recipients. Dotted lines indicate the lowest detectable neutralization titer (EC50 value of 41), and non-neutralizing titers (EC50 from 10–30) were arbitrarily set to 12 or 25 and boxed in the gray rectangle. Serum concentrations inhibiting 50% of the PsV infection (EC50 values) were calculated from median of triplicates.

For both vaccines, higher vaccine HPV type antibody levels were associated with more frequent and stronger cross-neutralization of non-vaccine HPV types. Ranked correlation of the neutralizing antibody levels to HPV16/45 (*r*_s_ = 0.53, 95% CI 0.37–0.66), and especially HPV18/45 (*r*_s_ = 0.61, 95% CI 0.47–0.72) was twice as strong in the bivalent/Finnish (Fig. 2a, b) than in the quadrivalent/Indian vaccine recipients (*r*_s_ = 0.34, 95% CI 0.16–0.49 and *r*_s_ = 0.28, 95% CI 0.10–0.44, Fig. 2c, d) even if the exact bivalent vaccine-induced HPV16 or HPV18 antibody levels were not always estimable. On the contrary, comparable correlation coefficients of HPV16/31 and HPV18/31 neutralizing antibodies were observed for both the bivalent vaccine (*r*_s_ = 0.66, 95% CI 0.53–0.75; and *r*_s_ = 0.68, 95% CI 0.56–0.77, Fig. 2e, f) and for the quadrivalent vaccine (*r*_s_ = 0.61, 95% CI 0.49–0.72; and 0.54, 95% CI 0.40–0.66, Fig. 2g, h). All ranked correlations of the neutralizing antibody levels are provided in the Supplementary Table.

## Discussion

We found significantly higher peak (Month 7) neutralizing HPV16 and HPV18 antibody levels in Finnish adolescent females, who received three doses of the bivalent vaccine, than in corresponding Indian quadrivalent vaccine recipients. We also found significantly increased seroprevalence to HPV33, HPV45, HPV52 and HPV58, and higher cross-neutralizing HPV31, HPV45 and HPV52 antibody titers in the bivalent/Finnish vaccine recipients as compared to the quadrivalent/Indian vaccine recipients.

Our findings on distinguishable cross-neutralizing antibody responses to HPV31/33/45/52/58 in adolescent women receiving the bivalent vaccine are in line with the earlier observations on the induction of wide cross-neutralizing peak antibody responses by the bivalent vaccine in adolescents [6]. The majority of the quadrivalent/Indian vaccine recipients had cross-neutralizing antibodies to HPV31 only. Moreover, strong correlation values were observed only between HPV16 and HPV31, and HPV18 and HPV31 in the quadrivalent/Indian vaccine recipients. Similar strong correlation between HPV16 and HPV31, HPV16 and HPV45, HPV18 and HPV31, and HPV18 and HPV45 neutralizing antibody levels was induced by the bivalent vaccine.

The cross-neutralizing antibody response induced by the two vaccines is largely the result of structurally comparable conformational epitopes shared among phylogenetically related HPV types.^10^ The fact that the bivalent/Finnish vaccine recipients presented stronger cross-neutralizing antibody responses, however, is intriguing. Probably a certain amount of antibodies differently induced by the presented adjuvants, a threshold level apparently between the bivalent and quadrivalent vaccine-induced ones, needs to be reached in order for the cross-neutralizing antibodies to be detectable. On the other hand, the low cross-reactive HPV45 antibody levels and the low correlation both between HPV16 and HPV45, and HPV18 and HPV45 antibodies in the quadrivalent vaccine recipients also suggest a qualitative difference. Presumably this might arise from better exposed cross-neutralizing epitopes on the bivalent vaccine VLPs vs. the quadrivalent vaccine VLPs, respectively produced in insect cells vs. yeast cells.^5^ Nevertheless, while the correlation data (Fig. 2 and Supplementary Table) implies that the same antibodies probably are able to mediate neutralization of two phylogenetically more or less related HPV types, this is particularly difficult to firmly state in polyclonal sera. It might be that boosting of low-level responses to alternate types occurs, but the type-specific antibodies are still narrowly reactive. The analysis of panels of neutralizing monoclonal antibodies would be required to conclusively explore this scenario.

Three main serological assays have been explored to validate immunogenicity of the current HPV vaccines in clinical trials and further monitoring the L1 VLP antibody responses in observational studies: antibody competition assays, L1 and L1-VLP-IgG binding assays and pseudovirion-based neutralization assays (PBNA), the latter of which should be considered the reference standard for assessing protective antibody responses induced by the vaccines, as it measures a pertinent biological antibody activity.^11^ The High-Throughput-Pseudovirion-Based Neutralization Assay (HT-PBNA) deployed here represents the most sophisticated and sensitive assay, which allows the simultaneous testing of larger numbers of samples by minimizing the complexity inherent to the manual neutralization assay, previously employed for HPV vaccine studies.^12^ Although in line with reports on differences in the HPV type-specific overall VE, the different GMT ratios presented here do not necessarily reflect differences between the two vaccines in protection against HPV infection. This strongly indicates that a head-to-head comparison of the sustainability of the neutralizing and cross-neutralizing antibody levels and protection induced by both vaccines is warranted.^9^

There are two relevant limitations to the evidence presented in our study. Firstly, two different cohorts were available for this independent study to investigate the peak neutralizing and cross-neutralizing antibody titers induced by the two HPV vaccines. It is arguable whether unknown environmental factors or even the different ethnicities related to each cohort here studied might have differentially influenced the antibody response. Despite of that, differences in bivalent and quadrivalent vaccine immunogenicity among adolescent women from geographically different regions, including Finland and India, were not reported by previous clinical trials.^9,13–16^ Secondly, given that the endpoint titers for many of bivalent/Finnish recipients were not accessed, as they reached values >180,000, the reported higher HPV16/18 antibody levels induced in the bivalent/Finnish recipients are under-estimations of the true difference in antibody response. Nevertheless, our conservative estimations oversee the need to investigate the cross-neutralizing properties of the two vaccines in further details.

The significantly different levels of quadrivalent and bivalent vaccine induced cross-neutralizing HPV31 antibodies are associated with reportedly comparable, high VEs against HPV31.^1^ On the other hand, the low levels of cross-neutralizing HPV33 and HPV45 antibodies confer moderate to high protection (>75% VE) only in the bivalent vaccine recipients. Our data also fit recent reports on VE from the Scotland, where significantly protective VEs against HPV31, HPV33, and HPV45 persistent infections, were observed among participants in a cervical cancer-screening program seven years after bivalent vaccination.^17,18^ Comparable VEs against HPV31, HPV45, HPV52, and HPV58 have also been reported in adolescent Japanese and Dutch girls vaccinated with the bivalent vaccine.^17,19,20^

The differences between the high neutralizing HPV6 antibody levels and high VE induced by the quadrivalent vaccine as compared to those induced by the bivalent vaccine (cross-neutralizing HPV6 antibody levels and low VE) are notable.^1,6^ It is surprising that many of the bivalent/Finnish recipients show cross-neutralizing antibodies to HPV6—a phylogenetically distant genotype to the vaccine incorporated ones. We think that, occasionally, the very low GMT to HPV6 (Fig. 2b) could have been triggered by natural HPV6/11 infection.

Correlates of protection provided by the vaccines, e.g., the threshold of antibody level that confers immunity against HPV infection, have been difficult to identify due to the high vaccine-specific VEs. Understanding the differences in the cross-neutralizing antibodies and cross-protection induced by the vaccines may ultimately help to identify protective vaccine-induced antibody levels. In view of recent studies on HPV VE upon a single-dose schedule, it may be critically important to follow-up the antibody titers over time by vaccine (bivalent vs multivalent) and by vaccine dose (1–3). The different adjuvants and number of doses are fundamentally linked to vaccine dissemination and uptake. This, however, was not the scope of our current study.

In conclusion, peak neutralizing antibody levels induced by the bivalent vaccine are 2- to 7-times higher in adolescent women for high-risk HPV types 16/18/31/33/45/52/58 than those induced by the quadrivalent vaccine. Notably, our data represent an initial preliminary study that requires confirmation in a head-to-head study throughout. Considering that the recently licensed nonavalent vaccine targets additional types associated with virtually all cervical cancer cases, independent post-licensure studies on protection and cross-protection are important to assure best vaccine effectiveness and health economic impact from the vaccine to be chosen for a national vaccination program.

## Methods

### Participants

The Finnish post vaccination (Month 7) serum samples were collected following an informed consent in conjunction with the randomized controlled PATRICIA trial.^2,21^ A total of 4,808 adolescent, female, PATRICIA trial participants received either the AS04-adjuvanted bivalent HPV16/18 vaccine (GlaxoSmithKline Biologicals, Rixensart, Belgium) or a control hepatitis A vaccine (GSK Biologicals). A random sample of 103 originally 16–17 year-old recipients of three doses (Months 0, 1, and 6) of the HPV16/18 vaccine was selected out of 2,409 HPV16/18 vaccinated participants.^2,21^ In this work, subjects from this cohort are referred as bivalent vaccine/Finnish recipients.

The Indian post vaccination (Month 7) serum samples were obtained from a multicentric trial, to investigate the effectiveness of two and three doses of quadrivalent HPV vaccine inducing non-inferior immune response in girls aged 10–18 years.^22^ The originally planned cluster-randomized trial initiated vaccination on 1 September 2009 and was converted to an observational study due to suspension of vaccination midway by a Governmental order on 8 April 2010 due to reasons unrelated to the study. We selected a random sample of 120 originally 15–18-year-old recipients of three doses of the HPV6/11/16/18 vaccine (Months 0, 2, and 6) out of 4,348 vaccinated female trial participants and representing all study sites. In this work, subjects from this cohort are referred as quadrivalent vaccine/Indian recipients.

### Ethics statement

The NCT01393470 trial was reviewed and approved by the Finnish National Ethical Review Board, ETENE/Tukija. The NCT00923702 trial was reviewed and approved by ethics review committee in India (Health Ministry Screening Committee of Government of India) and the IARC Institutional Review Board. Written informed consent from all participants were obtained at the time of the Finnish clinical trial. For the Indian clinical trial, written informed consent was obtained from one of the parents, or legal guardian, along with the assent of the participating girl.

### Pseudovirion-based neutralization assay

We determined neutralizing antibodies by a semi-automated and High-Throughput-Pseudovirion-Based Neutralization Assay (HT-PBNA) for HPV6/16/18/31/33/45/52/58.^23^ Briefly, the assay uses pseudovirions (PsV) made of HPV L1 and L2 proteins, which in turn encapsidate a Gaussia-luciferase reporter plasmid. After production in HEK293TT cells and purification by ultracentrifugation in an Optiprep gradient, these particles are able to bind to and enter in HeLaT cells, allowing transduction of the reporter gene. PsV infection and consequent Gaussia-luciferase activity are measured by luminescent reaction with coelentarazine as substrate. In the presence of neutralizing antibodies, however, PsV infection is blocked and transduction of the reporter gene is reduced or absent. Sera were analysed in serial dilutions ranging from 1:40 to 1:180,000 and serum concentrations inhibiting 50% of the PsV infection (EC50 values) were calculated from median of triplicates. EC50 values >40 (corresponding to 1.3 and 1.1 IU/ml for HPV16 and HPV18, respectively) were defined as neutralization seropositive and EC50 values >180,000 (corresponding to >5,818 and >5,019 IU/ml for HPV16 and HPV18, respectively) could not be differentiated further.

### Statistical analysis

HPV type-specific seroprevalence (percentage of neutralization-positive among all sera), geometric mean titers (GMT) (neutralization-positive sera only) with 95% confidence intervals and the two-tailed Spearman nonparametric correlation coefficients (*r*_s_) (all sera) were calculated using GraphPad Prism.

### Reporting summary

Further information on research design is available in the Nature Research Reporting Summary linked to this article.

## Supplementary information

Supplementary Table

Reporting Summary

## Data Availability

The authors confirm that the raw data of this HT-PBNA study were generated at the EMBL-DKFZ Chemical Biology Core Facility. Ranked correlation of the neutralizing antibody levels are available in the Supplementary Table. Additionally derived data supporting the findings of this study are available upon reasonable request to FCM (f.mariz@dkfz.de) and Noemi Bender (n.bender@dkfz.de) according to DKFZ data safety protection regulations.
