# Supplementary material for: Peak neutralizing and cross-neutralizing antibody levels to human papillomavirus types 6/16/18/31/33/45/52/58 induced by bivalent and quadrivalent HPV vaccines
Source: NPJ Vaccines. 2020 Feb 14;5:14. doi: 10.1038/s41541-020-0165-x (PMC7021830; doi:10.1038/s41541-020-0165-x)
Supplement: Supplementary file 1 — Supplementary Table [file 41541_2020_165_MOESM1_ESM.pdf]

Supplementary material

	Bivalent/Finnish				Quadrivalent/Indian					
	HPV16		HPV18		HPV16		HPV18		HPV6	
**HPV18**	0.83	(0.76-0.88)	-----	--------------	0.71	(0.61-0.79)	-----	--------------	-----	--------------
**HPV6**	0.02	(-0.17-0.21)	0.04	(-0.15-0.23)	0.74	(0.65-0.82)	0.76	(0.68-0.83)	-----	--------------
**HPV31**	0.66	(0.53-0.75)	0.68	(0.56-0.77)	0.61	(0.49-0.72)	0.54	(0.40-0.66)	0.49	(0.35-0.62)
**HPV33**	0.41	(0.23-0.56)	0.40	(0.22-0.56)	0.47	(0.32-0.60)	0.32	(0.14-0.47)	0.40	(0.23-0.54)
**HPV45**	0.53	(0.37-0.66)	0.61	(0.47-0.72)	0.28	(0.10-0.44)	0.34	(0.16-0.49)	0.23	(0.05-0.40)
**HPV52**	0.51	(0.34-0.64)	0.43	(0.25-0.58)	0.52	(0.37-0.64)	0.41	(0.24-0.55)	0.42	(0.26-0.56)
**HPV58**	0.38	(0.20-0.54)	0.45	(0.28-0.60)	0.16	(0.021-0.33)	0.19	(0.01-0.36)	0.15	(0.03-0.32)

Supplementary Table. Ranked correlation (Spearman coefficients (95%CI)) of the neutralizing and cross-neutralizing antibody levels to paired HPV types in bivalent/Finnish and quadrivalent/Indian vaccine recipients at month 7 post vaccination.
